# Bioactive Compounds in Functional Meat Products

**DOI:** 10.3390/molecules23020307

**Published:** 2018-01-31

**Authors:** Ewelina Pogorzelska-Nowicka, Atanas G. Atanasov, Jarosław Horbańczuk, Agnieszka Wierzbicka

**Affiliations:** 1 Department of Technique and Food Development, Faculty of Human Nutrition and Consumer Sciences, Warsaw University of Life Sciences (WULS-SGGW), Warsaw, Nowoursynowska Street 159c, 02-776 Warsaw, Poland; agnieszka_wierzbicka@sggw.pl; 2 Institute of Genetics and Animal Breeding, Polish Academy of Sciences, 05-552 Jastrzębiec, Poland; atanas.atanasov@univie.ac.at (A.G.A.); olav@rocketmail.com (J.H.); 3 Department of Pharmacognosy, University of Vienna, Althanstrasse 14, 1090 Vienna, Austria

**Keywords:** meat, functional food, additives, bioactive compounds, fortification

## Abstract

Meat and meat products are a good source of bioactive compounds with positive effect on human health such as vitamins, minerals, peptides or fatty acids. Growing food consumer awareness and intensified global meat producers competition puts pressure on creating new healthier meat products. In order to meet these expectations, producers use supplements with functional properties for animal diet and as direct additives for meat products. In the presented work seven groups of key functional constituents were chosen: (i) fatty acids; (ii) minerals; (iii) vitamins; (iv) plant antioxidants; (v) dietary fibers; (vi) probiotics and (vii) bioactive peptides. Each of them is discussed in term of their impact on human health as well as some quality attributes of the final products.

## 1. Introduction

Our perception of food has changed over the centuries. Originally, food was simply perceived as a means to obtain the necessary nutrients and energy for the body. Later on, due to the evolution of nutrition science, it began to be seen as a means of supporting adequate growth and development of the body. Nowadays food is also perceived as a key factor influencing the prevention of some diet-related diseases. Thus, a substantial effort in the food industry goes towards the improvement of food healthiness [1]. To increase the nutritional and health value of food, the addition of functional substances is applied. Primarily plants were thought to be the main sources of functional ingredients, but after further research, it has been reported that animal products also contain such substances. The idea of production of food with higher health impact is based on the fortification of original products with health-promoting ingredients. Meat and meat products seem to be a good matrix for designing novel products. The process of functional meat and meat product development can start in two stages: at the stage of animal nutrition and at the stage of its processing. Nonetheless, the addition of those ingredients may also change the appearance, taste, flavor and consistency of the original products. These changes are largely responsible for the rejection of many newly developed functional foods by consumers [2].

Therefore the aim of this paper is to overview the state of the art of the effect of bioactive compounds—both of plant and animal origin—used in animal nutrition as well as direct additives on different aspects of meat quality, including its nutritive value, physico-chemical and sensorial properties. Furthermore, we discuss the concentration of those constituents in products and their potential beneficial effects on human health.

## 2. Functional Compounds with Relevance for Meat Industry

### 2.1. Fatty Acids Quantitative and Qualitative Modifications

Overconsumption of saturated fatty acids (SFA) is a struggle for many developed countries while at the same time most of developing countries suffer from underconsumption of polyunsaturated fatty acids (PUFA) [3]. According to Dietary Guidelines for Americans (2015–2020) [4], daily intake of fats should not exceed 20–35% of total acquired energy. Furthermore not more than 10% of energy should be obtained in the form of saturated fatty acids. Polyunsaturated fatty acids consumption is recommend to constitute 5–10% energy from n-6 and 0.6–1.2% energy from n-3, with not less than 0.5% energy from *α-*linolenic acid (ALA) and 250 mg per day of eicosapentaenoic acid (EPA) and docosahexaenoic acid (DHA) [5]. In turn, most commonly recommended daily intake of conjugated linoleic acid (CLA) for adults is 0.8 gram per day [6]. Generally, the high contribution of animal fat in human diets linked with high cholesterol intake is believed to be associated with the occurrence of diet-related diseases such as coronary diseases [7]. Since meat contains a lot of fat and more than 40% is in saturated form, is advisable to change its quantity and quality creating new meat products of functional properties. In order to develop those products, three meat reformulation strategies have been developed: (i) reduction of total fat; (ii) reduction of total cholesterol intake and (iii) fatty acid profile modification.

Fat reduction is possible in three ways: increasing the CLA content in animals’ diet, using leaner raw parts of the carcass or diluting the fat density by the addition of water and low-caloric or non-caloric fat replacers such as gums, proteins or carbohydrates. However fat removal or its replacement may deteriorate the texture of the product or contribute to increased cooking loss [8]. Nonetheless, recent studies on the usage of guar gum as a fat substitute in low fat meat emulsions has revealed higher emulsion stability and cooking yield, and smaller percentages of metmyoglobin, fat oxidation products and protein carbonyls [9]. It is noteworthy that both high fat control and low-fat formulation containing 0.5% guar gum had comparable overall acceptability scores. On the contrary, the addition of okara, a soymilk production by-product, as a fat replacement in low-fat beef burgers improved tenderness, but decreased springiness, cohesiveness and deteriorated flavor in a dose-dependent manner [10]. Cholesterol content in meat should be also decreased because of its adverse effect on human health. Even though in 2015 cholesterol limits were abolished and there is no longer an upper limits on consumption (previously < 300 mg per day), it is still recommended in the 2015–2020 Dietary Guidelines for Americans [4] to eat as little dietary cholesterol as possible. Meat is quite reach source of cholesterol reaching of about 75–100 mg per 100 g of meat, depending on animal species. Thus different techniques of cholesterol reduction in meat are used. In the tissues of living animals a lower cholesterol content is observed after forage supplementation with copper in the case of poultry [11] or with CLA in pigs [12]. Cholesterol reduction is also possible after slaughter using for example cholesterol-reducing bacteria (*Eubacterium coprostanoligenes*) in fermented meat products (sausages) [13] or by meat and fat replacement with plant-based components. A third method of cholesterol depletion is meat enrichment with CLA. It has been proved that this component decreases cholesterol accumulation in LDL fraction possibly also in human organisms. Alternatively, the addition of phytosterols is recommended. Phytosterols are phytochemicals naturally occurring in plants and structurally similar to cholesterol. They have the ability to lower the cholesterol absorption in the small intestine [14]. Thus, aside from the possibility to eliminate cholesterol from meat, it is also possible to reduce its bioavailability.

Fatty acid composition in meat is unfavorable so it is essential to change it. Recently major attention was focused on CLA because of its proven biological activity in the prevention of obesity, cancer, diabetes, atherosclerosis and osteoporosis [15]. Conjugated linoleic acid is synthetized by the bacterium *Butyrivibrio fibrisolvens* in the rumen of ruminant animals, through the incomplete biohydrogenation of linoleic acid to stearic acid. In order to increase CLA in the meat of those animals a dietary supplementation with oils and oilseeds high in polyunsaturated fatty acids is recommended. Previous studies showed that safflower oil supplementation increased CLA in sheep by 134% [16] and sunflower oil increased CLA content in sheep meat of about 30–50% depending on the cut [17]. Higher CLA concentration in meat was observed also for animals fed with pasture rather than diet concentrates [18]. Furthermore an increase of CLA is observed in the meat of rumiant animals fed with CLA-protected additives. In the case of non-rumiant animals such as pork or chicken, meat enrichment is achieved by feeding animals with chemically synthetized CLA. However, increasing the CLA content in meat may change its textural and sensory properties. It might improve hardness, decrease lightness, decrease juiciness or contrary stabilize the color or lower purge loss depending on the animal species. Technologically CLA can be incorporated in meat by injections of commercial CLA isomers into muscles, or by direct addition to sausages or to patties. Noteworthily, heat treatment does not decrease CLA content in processed meat. Juarez et al. [19] made sausages with the addition of 6–7% of CLA, and after grilling the amount of those fatty acids remained unchanged. It is also important to supplement meat with other polyunsaturated fatty acids, and often oleic acid (ω-9) is especially used to change the profile of meat fatty acids because of its high stability. Furthermore linoleic acid and α-linolenic acid are also perceived as essential for humans. They have been proved to have cardiovascular and neuroprotective properties. The effective way to fortify meat with those fatty acids is to feed animals with oils or oilseeds [20]. Even more important from a dietary point of view is to add eicosapentaenoic acid and docosahexaenoic acid because they are most bioactive [21,22]. They are keys in proper fetal development, decreasing the incidences of allergies in infants, exerting antiplatelet and anti-inflammatory effects, and improving cognitive functions, etc. [23]. Unfortunately, these acids are also highly prone to oxidation, and for instance, DHA is far more unstable then oleic acid. Fish oils, which are a great source of EPA and DHA acids, are used as additives in the diet of non-ruminant animals [24]. However, after long-term storage or after thermal treatment the resulting meat was characterized by a slight increase in fish oil flavor [25]. The fatty acid composition of ruminants is hard to change because of the biohydrogenation of PUFA in the rumen. Some attempts was undertaken to use protected lipids, but the efficiency of fatty acid conversion was low and the costs of such intervention were high [26]. An alternative might be the usage of linseed or rapeseed oils as a source of linolenic acid (ALA) [27], which could be converted in the human body into DHA and EPA. Nonetheless, this is extremely inefficient and as much as five times more ALA needs to be absorbed to produce those acids in physiologically active form. Fish oil is also added directly to processed meat in encapsulated or pre-emulsified form, with no adverse effect on meat quality [28]. There are also a lot of other fatty acids of beneficial properties for human health, such as branched-chain fatty acids (BCFAs), which have promising potential for the creation of functional meat products. BCFAs are recognized for their antitumor properties against breast cancer cells [29].

### 2.2. Minerals

#### 2.2.1. Iron

Iron is a key component in human nutrition. This divalent metal has an important role in various physiological functions. It takes part in oxygen transport, the synthesis of enzymes, energy production and the regulation of immune functions [30,31]. Iron is also essential for proper brain development of the fetus and further in maintenance of neural connection and its activity. The recommended daily intake of iron for man is 8 mg, for non-pregnant women 18 mg [32] and for pregnant ones 27 mg. It was estimated that total net iron need for pregnant woman is almost 800 mg [33]. Those are relatively high doses, especially for pregnant women. Thus, approximately 30% of humans suffer from iron deficiency [34].

Food alone is not a sufficient source to meet those requirements. Because of that, iron fortification is applied, mainly in flours, but also in rice and cereals [35,36]. Those are the most commonly used matrices because of iron’s highly oxidant properties and specific metallic taste, but also because of the relatively low cost of those products. Nonetheless, taking into account that 22% of women living in developed countries suffer from iron deficiency [37], some other more expensive foods have started to be considered as possible matrices for iron fortification. Meat has started to be fortified with this metal but this causes some problems such as the fact its addition promotes rapid lipid oxidation and color deterioration [38]. This obstacle was overcame by usage of iron encapsulation in liposomes before direct addition to meats. An especially attractive medium for iron enrichment are patties, because of the great balance of heme iron, protein and saturated fat components [39]. A great source of iron may be also meats after thermal removal of water, which leads to condensation of this element. It was shown that after heat treatment there was 20–26% more iron in the products than in raw meat [40]. In case of animals’ diets, semi-intensive feeding systems should be applied for cattle in order to enrich beef with this mineral. Furthermore, the extended lifetime of animals contributes to higher doses of iron in meat [41]. Some meats in general could be perceived as functional foods because of the huge amounts of iron they contain. For instance emu meat contains 5 mg of iron per 100 g of wet muscle tissue and rhea meat 3.2 mg (for comparison chicken contains 0.4 mg and pork 0.36 mg [42]).

#### 2.2.2. Selenium

Selenium is an important trace element for human and animal health because is a part of selenoproteins and it regulates many physiological functions. Selenium’s mode of action is mainly based on its redox regulation role via neutralizing and removing free radicals. It is a key component in antioxidative defense, and is implicated in the regulation of the immune system, metabolism of thyroid hormones, male reproduction, prevention of pre-eclampsia, diabetes mellitus, cardiovascular diseases (CVD) and cancers [43]. The average selenium content in meat varies within a range from 13.9 µg (duck) up to 36.1 µg (pork) per 100 g of edible portion [44]. The recommended daily allowance (RDA) for adults (19–50 years old men and women) is 55 μg of Se per day. Daily intake below 30 μg results in deficiency symptoms such as juvenile cardiomyopathy (Keshan disease). The name of disease is taken from the Keshan region in north-east China where the soil is extremely selenium-deficient. The concentration of Se in soil varies depending on the organic matter content, soil type, texture and precipitation. Se-deficient soil, or soil contained poorly absorbed Se (selenite instead of selenate) leads to low accumulation of this mineral in plants, and in turn to low concentration in the tissues of animals that consume them. According to Stoffaneller and Morse [45] Se daily intake is insufficient in European and Middle Eastern countries. Thus the increase selenium concentration in human diets is of high importance. Selenium supplementation of animal feed, especially with organic selenium, increases its concentration in the meat of various animals [46,47]. In Korea, responding to market requirements, special premium chicken and pork brands called “Selen chicken” and “Selen pork” were produced, with 10 times more selenium content than regular pigs. Based on a study by Gjerlaug-Enger et al. [48], 175 g of meat obtained from pigs fed fodder supplemented with 0.4 mg Se/kg is sufficient to provide the daily recommended level for adults. The addition of selenium to animal feeds increases lipid oxidation stability, delays microbial growth [49], and increases the firmness and chewiness of chicken meat [50]. Pork meat enriched with organic selenium is characterized by lower drip loss, lower occurrence of pale meat and watery meat syndrome [46]. Selenium can also added directly to meat products. Garcia-Iñiguez et al. [51] added Se-yeasts at a concentration of 2 g/kg to fermented sausages. They established that a 50 g portion provided 100% of the recommended intake with no adverse effect on sensory evaluation.

#### 2.2.3. Calcium and Magnesium

According to the Dietary Guidelines for Americans 2005 [52] calcium and magnesium are under-consumed minerals. The Recommended Dietary Allowance (RDA) for calcium is 1000–1200 mg/day and for magnesium 320–420 mg/day, while calcium is absorbed with the diet at approximately 700 mg/day and magnesium 260–320 mg/day [53]. Calcium is the most abundant cation in the body and it is of importance for muscle functions, nerve transmission, intracellular transmission, vascular contraction and vasodilation [54]. In turn, magnesium is the fourth most commonly occurring cation in the human body, which is also a cofactor for many enzyme systems, takes part in energy metabolism and the synthesis of proteins and nucleotides [55]. Meat and meat products are not perceived as good sources of both those minerals. The average content of calcium in different species ranges from 7 mg (beef) to 17 mg (pork), while for magnesium it ranges from 19 mg (beef) to 25 mg (turkey) [44]. However the concentration in meat might be modulated by animal feed supplementation or direct addition of salt or brine to meats. Research shows that meat obtained from cattle fed a Ca-enriched diet was characterized by a higher concentration of this mineral [56]. Addition of magnesium to pigs’ fodder improved the color of meat, reduced drip, exudative and cooking losses, decreased fat oxidation and increased acceptability scores [57]. Salts of magnesium and calcium are mainly used in meat products for sodium chloride replacement. The limitation for the use of CaCl_2_ and MgCl_2_ salts in meat products are their negative effect on sensory quality because of their bitter taste, generation of off-flavors and slower penetration through muscle (divalent cations penetrate slower then NaCl). To compensate the unpleasant bitter taste, masking agents are used like sweet pepper, coriander, tomato, lime, parsley, etc. or salt mixtures (KCl, CaCl_2_ and MgCl_2_). Those techniques allow one to reduce salt levels in meat products by up to 40–50% without any negative impact on sensory attributes [58]. Addition of calcium and magnesium salts have also a positive effect on meat texture, especially calcium in activating calpain enzymes that lead to increased protein degradation what in turn increases tenderness [59], but also reduces emulsion stability, elasticity, cohesiveness and cooking yield [60].

#### 2.2.4. Zinc

Zinc is an essential trace element for human health. Although it is wildly abundant in Nature, its deficiency is an important worldwide health problem. Zinc has catalytic (over 100 enzymes are zinc-dependent), structural (it is important in protein and cell membrane maintenance) and regulatory (gene expression) functions in cells. Long-term insufficient intake of this mineral with the diet leads to anemia, diarrhea, pneumonia, disturbed brain development, abnormalities in fetal growth and development or at severe deficiencies to hypogonadism and dwarfism [61]. Zn-deficiency is recognized as a problem of developing countries, but is also reported among adults in UK or young adults in Spain. Even though meat and meat products are the main source of zinc in the human diet (20–40%), it is still added to animal feed as a supplement to increase its content even more and to improve the animals’ performance. However, results on the modification of content of Zn in meat from those animals are contradictory. According to Yang et al. [62] broiler feed supplementation with zinc resulted in a linear increase of zinc content in meat, while Norouzi et al. [63] did not note Zn content modification in those meats. The source of dietary zinc had an influence on feed intake and average daily weight gain. Inclusion in animal diet sources of organic zinc increased feed intake and body weight compared to inorganic zinc [64]. At the same time decreased fat oxidation, reduced cooking loss, increased crude protein content and increased total antioxidant capacity in chicken meat were observed [62]. The influence of zinc addition on meat color is unclear, with some studies reporting a yellowness increase in Zn-enriched meat [63], while others did not notice any effect [65].

#### 2.2.5. Iodine

Iodine is a key trace element for the synthesis of thyroid hormones. An insufficient iodine supply with the diet causes a number of health problems like skeletal deformations, diminished physical growth, goiter, decreased fertility and mental retardation collectively known as Iodine Deficiency Disorder (IDD). Pregnant women should take special care to consume a proper iodine supply because its deficiencies cause cretinism, increased prenatal death and infant mortality. Unfortunately IDD still remains a public health problem of high concern [66]. There have been also reported iodine deficiencies in pregnant womens’ diet all over the world [67]. Therefore some effort has been taken to fortify food with iodine. Increase in iodine content is possible by its addition to animal feed in order to accumulate it in tissues or by iodination of table salt. Excessive salt consumption is not recommended because of the risk of hypertension and atherosclerosis, so the iodine intake decreases even more [68], making animal products more important. The content of iodine in meats ranges from 0.59 mg per 100 g for red meat to 0.66 mg for poultry [69]. Addition of iodine concentrate to the drinking water of poultry resulted in an average 16–76% increase in meat [70]. Similarly a higher content of iodine was observed in meat from cattle fed forage with iodine concentrate, but this did not change dry matter intake, daily weight gain or slaughtering performance [71].

### 2.3. Vitamins

#### 2.3.1. Vitamin E

Vitamin E is a group of chemically similar compounds that includes tocopherols and tocotrienols. It possesses antioxidant ability to break the chain reactions of free radical formation. The most pronounced protective effect of vitamin E is observed against oxidation of the plasma lipoproteins and polyunsaturated fatty acid (PUFA) components of cell membranes [72]. Naturally vitamin E occurs in plants and cannot be synthetized de novo by animals. In order to introduce the vitamin into animal bodies, feed fortification is applied. Especially effective as a feed additive is α-tocopheryl acetate. Vitamin E deficiencies are rare in the human diet, however, its antioxidant activity makes it the most commonly added vitamin for animal feed. Even though meats were not initially the main source of vitamin E, dietary fortification has increased its content and nowadays it is considered as a moderate source (poultry ranks as the second dietary source of α-tocopherol) [1]. A number of studies have documented a correlation between feed fortification with vitamin E and its increased content in muscle cells with the duration of supplementation [73]. In grass finishing cattle vitamin E content increases three-fold in muscles (2.1 to 7.73 μg/g) in comparison to grain-fed animals (0.75 to 2.92 μg/g of muscle) [74]. Vitamin E fortification decreases lipid oxidation in chicken [75], cow [76], pork [27] and rabbit meat [77]. It was also observed that a high level of vitamin E reduced oxysterol formation during heat treatment [78]. Metmyoglobin formation was also inhibited by vitamin E addition to Hanwoo cattle forage, what improved meat color [79]. Additionally vitamin E decreases toughness (lowering shear force) [80], increases tenderness (improved collagen solubility) [81], increases water holding capacity and reduces rigor mortis (inactivation of phospholipase A2) [82] and has an impact on the profile of volatile compounds in meat [83,84]. Direct addition of vitamin E did not affect basal composition, lipid oxidation, microbial growth, cooking loss or texture of raw or cooked sausages [85]. Some studies report that heat treatment and storage duration did not change α-tocopherol retention in meat products [84], while others noted a significant decrease up to 58% [86].

#### 2.3.2. Vitamin D

Vitamin D status in the human body is of high importance because it is essential in calcium absorption and homeostasis. Insufficient intake of this vitamin leads to rickets in children and osteomalacia in adults. Recently possible roles of vitamin D or rather its deficiency in cardiovascular disease, type 1 diabetes, cancers, hypertension, rheumatoid arthritis, autoimmune conditions and Parkinson’s disease have been reported. Deficiency of vitamin D is a great health concern worldwide but in Europe it is described as a pandemic [87]. Daily recommended target intake should be around 10–20 µg/day (400–800 IU/day) assuming little or no exposure to sun, while it is shown that the actual intake is usually only about 3–7 µg/day (120–280 IU/day). Nowadays dairy products are fortified with vitamin D but it seems to be below the needs [88]. Feeding animals with vitamin D-enriched forage is used and its concentration increases in the tissues of supplemented animals [89]. Additionally, dietary supplementation positively affected color of meat (lower lightness L* and higher redness a*) [90], which is most important factor for consumer acceptance of meat during purchase. Feeding animals high levels of vitamin D prior to slaughter increased the tenderness of meat via activation of m-calpain [91]. However supranutritional doses of vitamin D within a short time prior slaughter did not improve this trait [92]. It has been also reported that meat of supplemented animals did not differ in sensory traits such as flavor, juiciness or off-flavor from control group [89].

#### 2.3.3. Vitamin C

Dietary supplementation of animals with vitamin C is widely studied. Frequently vitamin C is added to the feed in combination with vitamin E, because it protects vitamin E from oxidation processes via regeneration of α-tocopherol from tocopheryl radical [93]. Direct addition of vitamin C to meat is difficult because is not stable at the meat pH. Moreover it tends to stabilize the color of meat what is hazardous, masking potential changes such as microbiological activity and is considered adulteration for consumers. However when added to animal diet it accumulates in meat in a dose-dependent manner [94]. Results on the protective effect of vitamin C against lipid oxidation in meat are contradictory. According to Lo Fiego et al. [95] vitamin C reduced lipid oxidation, while Pion et al. [96] reported increased oxidation in supplemented meat. Decker and Xu [97] noted that vitamin C can act both as pro- and antioxidant depending on the dosed amount. Meat obtained from animals fed supplemented forage had improved marbling, firmness, texture and was more red and less pale then meat obtained from a control group [98]. Better texture profile of vitamin-C fortified meat (more tender) resulted from a higher level of collagen turnover that in muscle caused elevated collagen solubility.

#### 2.3.4. B-Group Vitamins

Consumption of sufficient amounts of B-group vitamins is essential for proper functioning of human body and particularly important are folate (B9) and vitamin B12. Folate is a key factor in the methylation process of nucleotides, which participate in tissue growth. Women with a low intake of folate during pregnancy are significantly more exposed to give birth to infants with neural tube defects, cancers, CVD and mental disorders [99]. In turn vitamin B12 is essential in DNA, RNA and protein synthesis. Deficiency of vitamin B12 leads to cognitive, and autonomic dysfunction, peripheral neuropathy and myelopathy [100]. Meats are considered as good source of vitamins from the B-complex group [101], for example portions of pork meat provide the daily required amount of thiamine. The average content of vitamin B12 in meats ranges from 0.4 µg (chicken) to 7.2 µg (rabbit). This vitamin is synthetized in the rumen of animals, and that is the reason red meat is a rich source of it [44]. However B-group vitamins are highly susceptible to degradation under heat treatment. Thus cooking may lead to great reductions of the amount of these vitamins, and there might be a need to balance this loss by direct addition of those vitamins to meat. Riccio et al. [102] showed that in fortified ham and burgers there were detected residues of vitamins B even after severe heat treatment (120 °C) and for control this was not the case. Noteworthily the addition of folic acid to ready-to-eat cooked meat products did not change the physicochemical properties of sausages and did not change acceptability [103].

### 2.4. Plant Antioxidants

#### 2.4.1. Polyphenols

Polyphenols is a huge group of phytochemicals comprising 8000 identified single compounds and this number is still rising. They are synthetized in plants as defense against various microorganisms and insects as well as to protect tissues from ultraviolet light and structural damage. Those chemicals are also responsible for the flavor, taste and color of plants. Well-known, rich sources of polyphenols are dark berries, vegetables, herbs, spices, teas, nuts, pomegranate, seeds and fruit and vegetable processing by-products (sweet potato peels, citrus peels, apple pomace, etc.) [104,105]. Numerous studies have reported a beneficial effect of polyphenols in the prevention of some diseases: ischemic heart diseases, Alzheimer’s disease, diabetes, liver disorders, allergies, bowel diseases and more [106,107,108,109,110]. Polyphenols have strong antioxidant properties related to free radical scavenging activity, chain-braking oxidation pathway activity and metal-chelating activity [111,112,113,114]. In addition the antimicrobial activities of phenolic compounds are well documented. It has been suggested that polyphenols destabilize the cell membranes of bacteria, inactivate enzyme activity, deregulate uptake of nutrients, interfering in nucleic acid and protein synthesis [115]. In response to consumers’ demands for foods free from drugs and synthetic additives, livestock producers have introduced new feeding strategies to increase the quality and nutritional value of meat. For that reason polyphenols are recently being utilized as animal’s feed additives. However, some authors suggests that polyphenols are poorly absorbed in the gut and absorbed polyphenols are extensively biotransformed and lose the beneficial properties. Recent studies show the opposite effects of the addition of polyphenols on the oxidative status of animal meat and its quality. Fortification of lambs’ diet with red wine extract [116], pigs with grape seed extract [117], cattle with tea catechins [118] or chicken with grape pomace [119] didn’t improve lipid oxidation stability or the quality of meat. Quite different results were obtained by Ranucci et al. [120] who fed pigs with plant extract mix composed of sweet chestnut wood extract and oregano essential oil (1:1). In the extract group higher glutathione peroxidase and glutathione reductase activity was observed. Furthermore fortification improved lipid stability and the color of meat. Similarly the addition of rosemary extract to lamb feed delayed lipid oxidation, color deterioration and microbiological spoilage [121], and supplementation of chokeberry pomace to lambs resulted in significant improvements of a range of blood and liver parameters linked to oxidative stress [122]. According to Luciano et al. [123] tannin supplementation of lambs improved color stability, increased total concentration of tannins in meat and elevated muscle antioxidant capacity. In turn chicken supplementation with gallic acid led to increased total phenolic content, DPPH reducing activity, ABTS^+^ reducing activity and improvement of the water holding capacity of meat [124]. In the case of polyphenols added to meat and meat products there are not so many contradictions in the results. The addition of grape extract to chicken meat [125], cherry and blackcurrant extract leaves to pork sausages [126], and citroflavan-3-ol to lamb patties [127] or pomegranate rind powder, pomegranate seed powder and kinnow rind powder to goat meat patties [128] inhibited lipid oxidation. Positive effects of polyphenol addition to meat or meat products on color [129] and sensory properties [130] has also been observed with no adverse effect on textural properties [131].

#### 2.4.2. Carotenoids

Carotenoids are a diverse group of colorants widely occurring in Nature. About 700 carotenoids of plant, algae, bacterial, moss and fern origin have been characterized. People and animals have no ability to synthetize carotenoids de novo, and they are mostly consumed from orangey vegetables and fruits or from green leaves. Carotenoids are very good quenchers of reactive oxygen species. Numerous studies provide a big body of evidence that carotenoids may reduce or even prevent some diseases such as cancer, cardiovascular diseases, age-related macular degeneration and other. Animal supplementation with carotenoids is applied in order to improve quality and nutritional value of meat. Addition of marigold extract rich in lutein and a small amount of zeaxanthin to broiler chicks diet improves the color of breast and thigh muscles, elevates antibody titres against Newcastle and influenza viruses and enhances growth performance [132]. Additionally, according to Wang et al. [133], marigold extract supplementation increases both yellowness (b*) and redness (a*) of thigh muscle, antioxidant capacity and decreases lipid oxidation and drip loss of chicken meat. Astaxanthin addition to chickens’ diet in the form of *Phaffia rhodozyma* increased the redness of meat in a dose dependent manner [134], while there were no dietary effect on pig meat color [135]. In turn, fucoxanthin administration increased antioxidant activity and improved the color of chicken meat. Dietary lycopene accumulated in liver and decreased cholesterol content in chicken meat [136]. Direct addition of both fucoxanthin and lutein to meat products decreased their lightness and increased redness [137]. Nonetheless, there are also some drawbacks to the increased content of carotenoids in meat. Such situations are noticed for grass-fed cattle. The content of carotenoids in the meat of these animals is higher but it is also associated with a change in the color of fat to yellow, which is unattractive for consumers [138]. Recently a vast amount of scientific attention is focused on the utilization of vegetable by-products as carotenoid-rich sources used to meat processing. Among them tomato by-products are of high interest. A part of the influence exerted by the addition of tomato by-products on meat quality is linked to simultaneous fiber addition, especially in the case of textural properties. For instance addition of tomato peel to beef hamburgers, even though increased redness of meat at the same time also increased its hardness [139]. Other researchers also noted an influence of tomato by-products on a decrease of lipid oxidation [140] and rancidity [141].

### 2.5. Dietary Fibers

According to the American Association of Cereal Chemists (AACC) “dietary fiber is the remnants of the edible part of plants and analogous carbohydrates that are resistant to digestion and absorption in the human small intestine with complete or partial fermentation in the human large intestine. It includes polysaccharides, oligosaccharides, lignin and associated plant substances. Dietary fiber exhibits one or more of either laxation (fecal bulking and softening; increased frequency; and/or regularity), blood cholesterol attenuation, and/or blood glucose attenuation” [142]. The recommended daily intake of dietary fiber is 20–30 g/day, but a vast majority of adults and children (>90%) do not meet those thresholds. This is of high health concern because a number of research reports shows that dietary fiber provides many health benefits: helps to reduce weight, stimulate immune system and decreases risk of development CVD, glycaemia, gastrointestinal disorders [143]. Thus there is a need to enrich food with dietary fiber. However, not only nutritional value is taken into consideration when using dietary fibers as functional additives but also technological properties. Fortification of finishing pigs’ diet with fiber had little impact on growth performance of animals but increased concentration of volatile fatty acid [144]. Addition of dietary fiber increased water-holding capacity and improved cooking properties of beef burgers [145,146]. It also changes color of meat depending of the fiber type used as supplement. Grape antioxidant dietary fiber tended to increase redness of raw and cooked chicken hamburgers [147]. In turn hazelnut pellicle addition decreased all color parameters measured in low-fat beef burgers [145]. Fiber addition changes also textural properties of meat, increasing hardness [148]. Generally, meat with smaller amounts of added fiber is more preferred by consumers [149].

### 2.6. Probiotics

Antibiotics are widely used in animal agriculture. It was estimated that 80% of antibiotics sold in United States is applied in animal breeding [150]. Overuse of antibiotics is dictated by preventive purposes against animal diseases or by the usage as growth factor. However extensive use of those has led to an emerging problem of antibiotic resistance of pathogenic bacteria, the serious threat for human health. Thus it is vital to eliminate common utilization of antibiotics in animal production. As an alternative for antibiotics the addition of probiotics to animal forage had been proposed [151]. Probiotics, in accordance with the World Health Organization definition [152] are “live microorganisms which, when administered in adequate amounts, confer a health benefit on the host”. Various beneficial effects of probiotics on animal health was documented such as modulation of the immune system, microbial balance in gut and neutralization of adverse effect of intestinal pathogenic bacteria. Additionally, probiotics has also proven beneficial effects in prevention of different types of cancers, obesity, type 2 diabetes, insulin resistance, non-alcoholic fatty liver disease and allergic diseases [153,154]. Even though probiotics exert positive effects on animal and human health, the impact on animal performance as well as meat and carcass quality is controversial. Some authors show that the addition of probiotics to feed has no effect on broiler performance, but increased the water holding capacity of meat [155]. Others noted decreases in color lightness but did not observed differences in water holding capacity, cooking loss and shear force of meat obtained from probiotic-fed broilers and control groups [156]. On the contrary, Zhou et al. [157] reported that the addition of 200 mg/kg and 400 mg/kg of probiotics decreased values of drip loss, cooking loss and shear force. However, they observed higher muscle weight and thickness of muscle fibers in those groups. Similarly, Abdullaa et al. [158] reported beneficial effect of probiotics on drip loss and cooking loss. Additionally they observed lower fat value for probiotic-fed chicken, while Novak et al. [159] observed higher content of abdominal fat and higher conductivity of meat after probiotic supplementation. It even unfavorably changed the ratio of LDL to HDL cholesterol in animal’s blood. In turn, probiotic administration in breeding pigs’ resulted in positive changes of the fatty acid profile in meat. Animals from the probiotic group were characterized by higher content of monounsaturated and polyunsaturated fatty acids in comparison to control [160]. The use of probiotics in animal feed can also result in positive changes of meat flavor characteristics [133]. Probiotics are also added directly in meat products. An especially attractive medium for the use of probiotics are fermented sausages, since they do not need heat treatment and they provide protection for probiotic bacteria in the human gut.

### 2.7. Bioactive Peptides

Bioactive peptides are short polymers composed of about 2–20 amino acids linked by peptide bonds. In food they are inactive, being a part of precursor proteins. As a result of food processing (fermentation, aging, enzyme treatment) or during digestion they are released. Active peptides can affect many physiological functions in human body like antioxidant, antihypertensive, antimicrobial, anticancer, antithrombotic, anti-diabetic, immunomodulating and probiotic [161]. They are safe and obtained from inexpensive raw materials, often from waste using relatively cheap technology, which overall makes them promising functional food ingredients. One of the best-studied examples of bioactive peptides are peptides possessing the ability to inhibit angiotensin I-converting enzyme (ACE) [162]. This enzyme is responsible for creating angiotensin II, which constricts arteries, resulting with higher blood pressure. Thus bioactive peptides inhibiting ACE have antihypertensive properties. It has been proved that pharmaceutical ACE inhibitors are effective in decreasing mortality linked to myocardial infarction by 11% up to 23% for risk population [163,164]. However, the use of artificial ACE-inhibitors is fraught with numerous side effects (skin rashes, cough, angioedema) as opposed to naturally acquired [165]. In order to introduce higher doses of bioactive peptides to the human diet a few strategies can be applied. The first strategy is based on introduction of some fermented meat products into one of the daily meals. The second strategy is to add into foods specific proteins as starters for the production the ACE inhibitor peptides. However this strategy is hard to implement because huge doses of proteins need to be introduced. The third strategy involves the direct addition of bioactive peptides into food. With this method there are also some limitations. The major obstacle is that they significantly impair the sensory properties of the product, making it bitter. To overcome this, peptides of animal origin are used (hydrolysates of meat or gelatin) as less bitter then from other sources [166]. Furthermore, encapsulations of bioactive peptides with liposomes or water in oil emulsions are applied. Those methods allow the possibility to introduce to the market new meat products of functional properties.

## 3. Summary

Generally there is an increasing trend of using functional bioactive compounds in meat production. There is no doubt that those components can exert a significant effect on human health maintenance. Nonetheless, the choice of substance type and its quantity depends on the product meant to be obtained. More specifically, an important issue is whether the product will be processed and if so, what kind of processing will be applied. Equally important in functional meat products design is also the consideration of dietary deficiencies of specific substances noted in various human populations. Summarizing, the process of creating new meat products of functional properties is complex and depends not only on the impact of applied functional ingredients on nutritional value, but also on the final quality of the meat.
